# Regulation of Inflammation by IL-17A and IL-17F Modulates Non-Alcoholic Fatty Liver Disease Pathogenesis

**DOI:** 10.1371/journal.pone.0149783

**Published:** 2016-02-19

**Authors:** Daniel A. Giles, Maria E. Moreno-Fernandez, Traci E. Stankiewicz, Monica Cappelletti, Stacey S. Huppert, Yoichiro Iwakura, Chen Dong, Shiva K. Shanmukhappa, Senad Divanovic

**Affiliations:** 1 Division of Immunobiology, Cincinnati Children’s Hospital Research Foundation and the University of Cincinnati College of Medicine, Cincinnati, Ohio, United States of America; 2 Division of Gastroenterology, Hepatology and Nutrition, Cincinnati Children’s Hospital Research Foundation and the University of Cincinnati College of Medicine, Cincinnati, Ohio, United States of America; 3 Research Institute for Biomedical Sciences, Tokyo University of Science, Noda, Chiba, Japan; 4 Department of Immunology, MD Anderson Cancer Center, Houston, Texas, United States of America; 5 Division of Pathology and Laboratory Medicine, Cincinnati Children’s Hospital Research Foundation and the University of Cincinnati College of Medicine, Cincinnati, Ohio, United States of America; INRA, FRANCE

## Abstract

Non-alcoholic fatty liver disease (NAFLD) has become the most common chronic liver disease worldwide. While it is well-accepted that inflammation is central to NAFLD pathogenesis, the immune pathway(s) orchestrating disease progression are poorly defined. Notably, IL-17RA signaling, via IL-17A, plays an important role in obesity-driven NAFLD pathogenesis. However, the role of the IL-17F, another IL-17RA ligand, in NAFLD pathogenesis has not been examined. Further, the cell types expressing IL-17RA and producing IL-17RA ligands in the pathogenesis of NAFLD have not been defined. Here, IL-17RA^-/-^, IL-17A^-/-^, IL-17F^-/-^ and wild-type (WT) mice were fed either standard chow diet or methionine and choline deficient diet (MCDD)—a diet known to induce steatosis and hepatic inflammation through beta-oxidation dysfunction—and hepatic inflammation and NAFLD progression were subsequently quantified. MCDD feeding augmented hepatic IL-17RA expression and significantly increased hepatic infiltration of macrophages and IL-17A and IL-17F producing CD4^+^ and CD8^+^ T cells in WT mice. In contrast, IL-17RA^-/-^, IL-17A^-/-^, and IL-17F^-/-^ mice, despite increased steatosis, exhibited significant protection from hepatocellular damage compared to WT controls. Protection from hepatocellular damage correlated with decreased levels of hepatic T-cell and macrophage infiltration and decreased expression of inflammatory mediators associated with NAFLD. In sum, our results indicate that the IL-17 axis also plays a role in a MCDD-induced model of NAFLD pathogenesis. Further, we show for the first time that IL-17F, and not only IL-17A, plays an important role in NAFLD driven inflammation.

## Introduction

Non-alcoholic fatty liver disease (NAFLD) has become the most common chronic liver disease worldwide [1]. NAFLD represents a spectrum of disorders ranging from steatosis (benign fat accumulation) to nonalcoholic steatohepatitis (NASH) and cirrhosis [1]. Although the etiology of NAFLD is multifactorial and remains largely enigmatic, it is well accepted that inflammation is a central component of NAFLD pathogenesis [2]. However, the critical immune mediators regulating NAFLD progression remain incompletely defined. Currently, only limited therapies for NAFLD exist [2]. A better understanding of the immune pathways driving NAFLD may consequently help in the development of novel therapeutics. While a variety of immune mediators have been associated with NAFLD pathogenesis, recent reports highlight an important role for the IL-17 axis in mediating disease development and progression [3–5].

The IL-17 axis includes a small family of proinflammatory cytokines: IL-17A, IL-17B, IL-17C, IL-17D, IL-17E, and IL-17F [6]. IL-17A, the best described and characterized family member, is produced largely in the skin, mucosal tissues and liver [5, 6]. Notably, IL-17A levels correlate with a variety of human hepatic disorders, including alcoholic liver disease [7], primary biliary cirrhosis [8], chronic hepatitis B and C [9, 10], liver transplant rejection [11] and hepatocellular carcinoma [12]. Additionally, IL-17A drives multiple hepatic diseases in murine models, including Con A-induced hepatitis [13] and carbon tetrachloride-induced liver injury [14]. Another IL-17 family member, IL-17F, does not have a currently defined role in hepatic disorders. Of interest, IL-17A and IL-17F signal through a heterodimeric receptor consisting of both IL-17RA and IL-17RC subunits [6]. IL-17RA is expressed ubiquitously, including expression by resident liver cells [7, 15]. In the context of NAFLD, IL-17RA signaling is critical in promoting NAFL to NASH progression [3]. Specifically, when fed obesogenic diet, IL-17RA^-/-^ mice exhibit increased obesity and hepatic steatosis, but are protected from hepatocellular damage [3].

However, the critical IL-17RA signaling ligands (IL-17A and IL-17F), during NAFLD remain under-defined. Given that: (a) IL-17A has a causal role in multiple models of liver injury; (b) IL-17F shares a receptor with IL-17A; and (c) IL-17RA mice are protected from obesity-induced NASH, we hypothesized that both IL-17A and IL-17F signaling through IL-17RA is critical to NAFLD progression.

Thus, here we employed methionine and choline deficient diet (MCDD), a diet that impairs hepatic beta oxidation; thus, driving NAFLD progression, (e.g. steatosis, hepatocellular damage, inflammation and fibrosis). Here we show for the first time that WT mice placed on MCDD exhibit increased hepatic infiltration of IL-17A and IL-17F producing CD4^+^ and CD8^+^ T cells, along with increased hepatic IL-17RA expression by non-hematopoietic cells. Further, we show that deficiency of IL-17A, IL-17F or IL-17RA results in increased steatosis, but reduced steatohepatitis when fed MCDD. This uncoupling of steatosis from steatohepatitis is characterized by decreased hepatocellular damage, decreased T cell and macrophage recruitment to the liver and decreased production of proinflammatory mediators associated with NAFLD progression. In sum, we show a novel role for IL-17F signaling in NAFLD pathogenesis along with a role for IL-17RA and IL-17A in MCDD-driven NAFLD, and provide initial evidence for the critical IL-17RA expressing cells and the cells producing its signaling ligands.

## Materials and Methods

### Mice

All mice were male, on a C57BL/6 background and were matched for nicotinamide nucleotide transhydrogenase (Nnt) mutation status [16]. Mice were housed in a specific pathogen-free facility, fed food and water ad libitum, and provided care in accordance with the Guide for the Care and Use of Laboratory Animals. All studies were approved by the Cincinnati Children’s Hospital Medical Center’s Institutional Animal Care and Use Committee. WT mice, IL-17RA^-/-^ mice, IL-17A^-/-^ mice, and IL-17F^-/-^ mice were bred at CCHMC. WT mice were originally obtained from Jackson, while IL-17RA^-/-^ mice were obtained from Amgen. IL-17A^-/-^ and IL-17F^-/-^ mice were kindly provided by Drs. Iwakura and Dong, respectively.

### NAFLD model

Beginning at 8 weeks of age, mice were fed either methionine and choline deficient diet (MCDD; Research Diets #A02082002B; 16% Protein, 63% Carbohydrate and 21% Fat kcal/gram) or chow diet (LAB Diet #5010; 29% Protein, 13% Fat and 58% Carbohydrate kcal/gram) ad libitum for 4 weeks. For all studies, animal body weight and food consumption was quantified weekly. Fresh food was provided on a weekly basis.

### Liver triglycerides

A pre-weighed quantity of frozen mouse liver tissue was placed in 10 μL of Homogenization Buffer (50 mM Tris, 150 mM NaCl, 1 mM EDTA, 1 mM PMSF) per mg of liver tissue. A 1/8” steel bead was added to each sample, and samples were next dissociated using a TissueLyser (Qiagen) for 3 minutes at 30 Hz. Following 20 minutes of incubation on ice, each sample was diluted 1:10 in homogenization buffer and added to a 96 well clear flat bottom plate (Costar) containing 200 μL of Triglyceride Reagent (Pointe Scientific). Standards (Pointe Scientific) were prepared according to manufacturer’s instructions. Hepatic triglycerides were quantified at 500–520 nm using Molecule Devices vmax and Kinetic Microplate Reader and SoftMax Pro v5 software.

### Hepatocellular damage

Alanine transaminase (ALT) levels were quantified from 10 μL of mouse serum per sample in a 96 well flat-bottom plate (Costar) and a BioTek Synergy 2 Multi-Mode Microplate Reader with Gen5 ver. 2.00 software. Catatrol I and II (Catachem Inc.) were used as controls and were prepared according to manufacturer instructions. ALT buffer was prepared by mixing ALT Activator Reagent and ALT sample Diluent Reagent (Catachem Inc.) and 200 μL was subsequently added to each sample. Starting at time zero, absorbance was recorded at 340 nm and 37°C once per minute for 5 minutes and blank value was subtracted from all samples. The ALT concentration (U/L) was subsequently calculated using the equation ALT U/L = (OD/min x 205) / (6.22 x 0.2 x0.005), where OD = change in absorbance.

### Flow cytometry

Single cell suspensions were derived from hepatic digestion as described and stained with directly conjugated monoclonal antibodies or isotype controls (all eBioscience) [3, 17]. To determine IL-17A and IL-17F production, total single cells were stimulated for 5 h with 50 ng/ml PMA (Sigma-Aldrich, St. Louis, MO) and 1 μg/ml Ionomycin (Calbiochem), in presence of brefeldin A (10 μg/mL, Sigma-Aldrich). Subsequently, flow cytometry was used to enumerate immune cell populations as described [18–20]. Briefly, cells were treated with FACS fix buffer (BD Biosciences) for 15 minutes, washed and incubated in PBS supplemented with 2% FBS and were subsequently stained with Live/Dead stain (Zombie UV Dye: Biolegend) and with directly-conjugated monoclonal antibodies to CD45-AF700 (104), TCRβ-PE (H57-597), CD4-APCef780 (GK1.5), CD8-ef450 (53–6.7), CD11b-PE (M1/70), CD11c-Percp (N418), F4/80-APCef780 (BM8), Ly6C-Percp (HK1.4) Gr1-FITC (RB6-8C5), NK1.1-PECy7 (PK136), B220-ef450 (RA3-6B2), IL-17RA-APC (PAJ-17R), TNF-BV650 (MP6-XT22), IL-17A-Percp (17B7) and IL-17F-PE (18F10) [all antibodies from e-Bioscience] for 30 minutes. Flow cytometry data were then collected using a LSR Fortessa (BD) flow cytometer and analyzed using FlowJo X software (vX0.7).

### Serum triglycerides

10 μL of serum was added to a 96 well clear flat bottom plate (Costar) containing 200 μL of Triglyceride Reagent (Pointe Scientific). Standards (Pointe Scientific) were prepared according to manufacturer’s instructions. Serum triglycerides were quantified at 500–520 nm using Molecule Devices vmax and Kinetic Microplate Reader and SoftMax Pro v5 software.

### Serum cholesterol

Serum was initially diluted 1:10 in saline and 25 μL of diluted serum or standard was then added to a 96 well clear flat bottom plate (Costar) containing 100 μL of Infinity Cholesterol Liquid Stable Reagent (Thermo Scientific). Standards (Thermo Scientific) were prepared according to manufacturer’s instructions. Serum cholesterol was quantified at 500–520 nm using Molecule Devices vmax and Kinetic Microplate Reader and SoftMax Pro v5 software.

### Liver mitochondrial isolation

Section of a large liver lobe was isolated and finely minced in 4°C liver isolation buffer (LIB; containing 210mM D-mannitol, 70mM sucrose, 5mM 4-(2-hydroxyethyl)-1-piperazineethanesulfonic acid 1mM ethylene glycol tetraacetic acid (EGTA) and 0.5% fatty free acid bovine serum albumin (BSA; MP Biologicals; pH 7.4), homogenized in a glass/glass homogenizer (Kimble-Chase) and centrifuged at 700 x g for 10 minutes at 4°C. Supernatant was collected and centrifuged at 10,000 x g for 10 minutes at 4°C. The pellet was re-suspended in LIB and centrifuged at 10,000 x g for 10 minutes at 4°C. To increase mitochondria purity, the pellet was resuspended in LIB without BSA and centrifuged at 10,000 x g for 10 minutes at 4°C. Mitochondria containing pellet was subsequently re-suspended in Mitochondrial Assay Solution (MAS-1 buffer; pH 7.4) containing 70mM sucrose, 220mM D-mannitol, 5mM potassium di-hydrogen phosphate (Fisher Scientific), 5mM magnesium chloride (Fisher Scientific), 2mM HEPES (ICN Biomedicals), 1mM EDTA (Sigma) and 0.2% fatty free acid BSA (MP Biologicals) and mitochondrial protein concentration was determined with a bicinchoninic acid protein assay kit (BCA; Thermo Scientific Pierce) according to manufacturer’s instructions.

### Mitochondrial substrate utilization quantification

An XF Analyzer (Seahorse Bioscience) was used to measure bioenergetics of mitochondria isolated from liver. Briefly, XF24 extracellular flux assay cartridge (Seahorse Bioscience) was hydrated overnight at 37°C according to manufacturer’s instruction. Isolated mitochondria (10 μg per well) were plated in supplemented MAS-1 buffer on a polyethylenimine pre-coated XF Cell Culture Microplate, centrifuged at 2200 x g for 20 minutes at 4°C and incubated at 37°C in a non-CO_2_ incubator for 10 minutes. For mitochondrial uncoupling, 5mM succinate, 5mM succinate/1mM adenosine dipshosphate (ADP), 2μM oligomycin and 4μM carbonyl cyanide p-trifluoromethoxyphenylhydrazone (FCCP) were sequentially injected and mitochondrial oxygen consumption rate was quantified.

### Histology

Hematoxylin and eosin staining was performed on 5 μm sections from the paraffin-embedded tissue blocks for conventional light microscopy analysis. For CD68 staining, the liver sections were de-paraffinised and immunostained with rabbit anti-CD68 antibody (Abcam) using the automated Ventana immunostainer, according to manufacturer's recommendation. For CD3 staining, the liver sections were de-paraffinised and immunostained with rabbit monoclonal anti-CD3 antibody (Ventana Medical Systems, Inc) using the automated Ventana immunostainer, according to manufacturer's recommendation. For Sirius Red staining, liver sections were de-parrinised and stained using standard protocol.

### qRT-PCR

Tissue samples were homogenized in TRIzol (Invitrogen) using a TissueLyser (Qiagen) set at 30Hz for 5 minutes and 1/8” diameter stainless steel beads (McMaster-Carr). RNA was extracted according to manufacturer instructions, resuspended in DEPC-treated water and quantified using a Nanodrop ND-1000. One microgram of RNA was treated with 1U amplification grade DNase I (Invitrogen) and reverse-transcribed using oligo-dT and random hexamers in presence of 200U Superscript II reverse transcriptase (Invitrogen). The cDNA was subsequently treated with RNAse H, diluted in DEPC-treated water and subjected to qPCR analysis using Light Cycler 480 II (Roche). Sybr Green I Master mix (Roche) and the following primers pairs were used: IL-17RA For CATCACACTCATCGCCATTC Rev TGCCATTGATTTTGGAGTCA SCD-1 For GAGGCCTGTACGGGATCATA Rev CAGCCGAGCCTTGTAAGTTC; SREBP-1c For CTGTCTCACCCCCAGCATAG Rev GATGTGCGAACTGGACACAG; KLF15 For ACAACTCATCTGAGCGGGAA Rev CACAAATGCACTTTCCCAGG; PPARα For CATGGGGAGAGAGGACAGA Rev AGTTCGGGAACAAGACGTTG; LIPE For TCTCGTTGCGTTTGTAGTGC Rev ACGCTACACAAAGGCTGCTT; CXCL10 For CCTATGGCCCTCATTCTCAC Rev CGTCATTTTCTGCCTCATCC; CCL2 For AGATGCAGTTAACGCCCCAC Rev TGTCTGGACCCATTCCTTCTTG; CCL22 For TGGAGTAGCTTCTTCACCCA Rev TCTGGACCTCAAAATCCTGC; TNF-α For CCAGACCCTCACACTCAGATCA Rev CACTTGGTGGTTTGCTACGAC; GMCSF For GCGACACGAGGATGAAGCA Rev AACCTCCTGCACGTCACTCC; CXCL1 For ACCCAAACCGAAGTCATAGC Rev TCTCCGTTACTTGGGGACAC; Beta-actin For GGC CCA GAG CAA GAG AGG TA Rev GGTTGGCCTTAGGTTTCAGG. mRNA expression of each gene was compared to Beta-actin expression (endogenous house keeping gene control).

### Statistical analysis

Data were analyzed employing unpaired student’s t test. All values are represented as means + SE and graphed with Prism 5a software (GraphPad Software, Inc.).

## Results

### MCDD-driven NAFLD is associated with increased IL-17 axis activation

**Fig 1:**
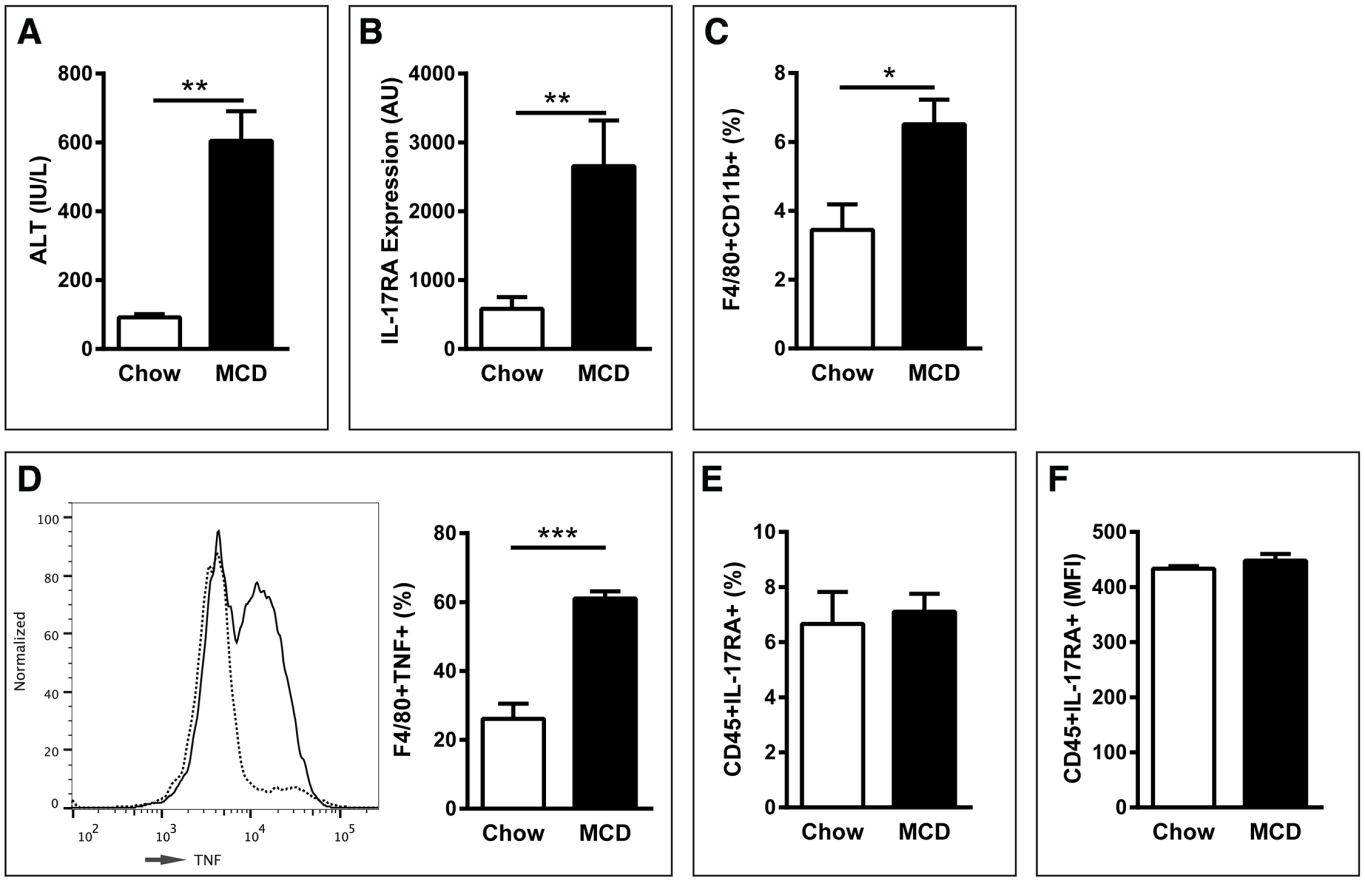
MCDD-driven NAFLD augments resident liver, but not immune cell IL-17RA expression and is associated with increased hepatic macrophage recruitment and activation. 8 week old male C57BL/6 mice (*n* = 4/condition) were placed on MCDD or chow diet for 4 weeks. (**A**) Hepatic IL-17RA mRNA expression. (**B**) Percent of hepatic immune cells expressing IL-17RA. (**C**) Mean fluorescent intensity of hepatic immune cells expressing IL-17RA. (**D**) Percent of hepatic immune cells (CD45^+^) expressing CD11b and F4/80. (**E**) Percent of hepatic macrophages (F4/80+) producing TNF. Dotted line = chow fed. Solid line = MCDD fed. (**F**) Serum ALT levels. AU = Arbitrary Units, as compared to beta actin. Data represent means + SE from a single experiment. Student *t* test *P < 0.*05*, **P < 0.01, ***P < 0.001.

Obesity-induced NAFLD progression is associated with increased systemic and hepatic IL-17A expression [3], however whether IL-17 axis plays a role in other models of NAFLD has not been examined. Here, we hypothesized that the role of IL-17 axis in NAFLD progression is conserved across models and, specifically, that MCDD feeding would exacerbate IL-17 axis activity, and specifically increase IL-17RA, IL-17A and IL-17 expression. As shown in Fig 1A, WT mice fed MCDD exhibit significantly increased hepatic IL-17RA expression compared to WT mice fed chow diet. Interestingly, the increase in IL-17RA expression was likely conserved to liver resident cells, as it was independent of differential percent and mean fluorescent intensity (MFI) by liver hematopoietic cell (CD45+) expression (Fig 1B and 1C). Further, in depth analysis of IL-17RA expression by innate immune cells, including macrophages, dendritic cells and neutrophils also revealed similar expression levels on either diet (data not shown). This increased expression of IL-17RA correlated with a significant increase in hepatic CD11b+F4/80+ macrophage infiltration and a trend towards increased macrophage skewing to a proinflammatory phenotype [21] as quantified by macrophage Ly6C^hi^ expression (Fig 1D and data not shown). Notably, MCDD-driven increase in macrophage infiltration directly correlated with robust augmentation in macrophage specific TNF production (Fig 1E). Importantly, proinflammatory macrophage infiltration has been associated with induction of hepatic inflammation and hepatocellular damage[22, 23]. Further, as previously demonstrated, MCDD feeding, even short term (4 weeks), robustly upregulated hepatocellular damage as quantified by serum alanine transaminase (ALT) levels (Fig 1F)[24]. These data suggest that MCDD feeding alters expression of IL-17RA, something that correlates with increased macrophage infiltration to the liver.

**Fig 2:**
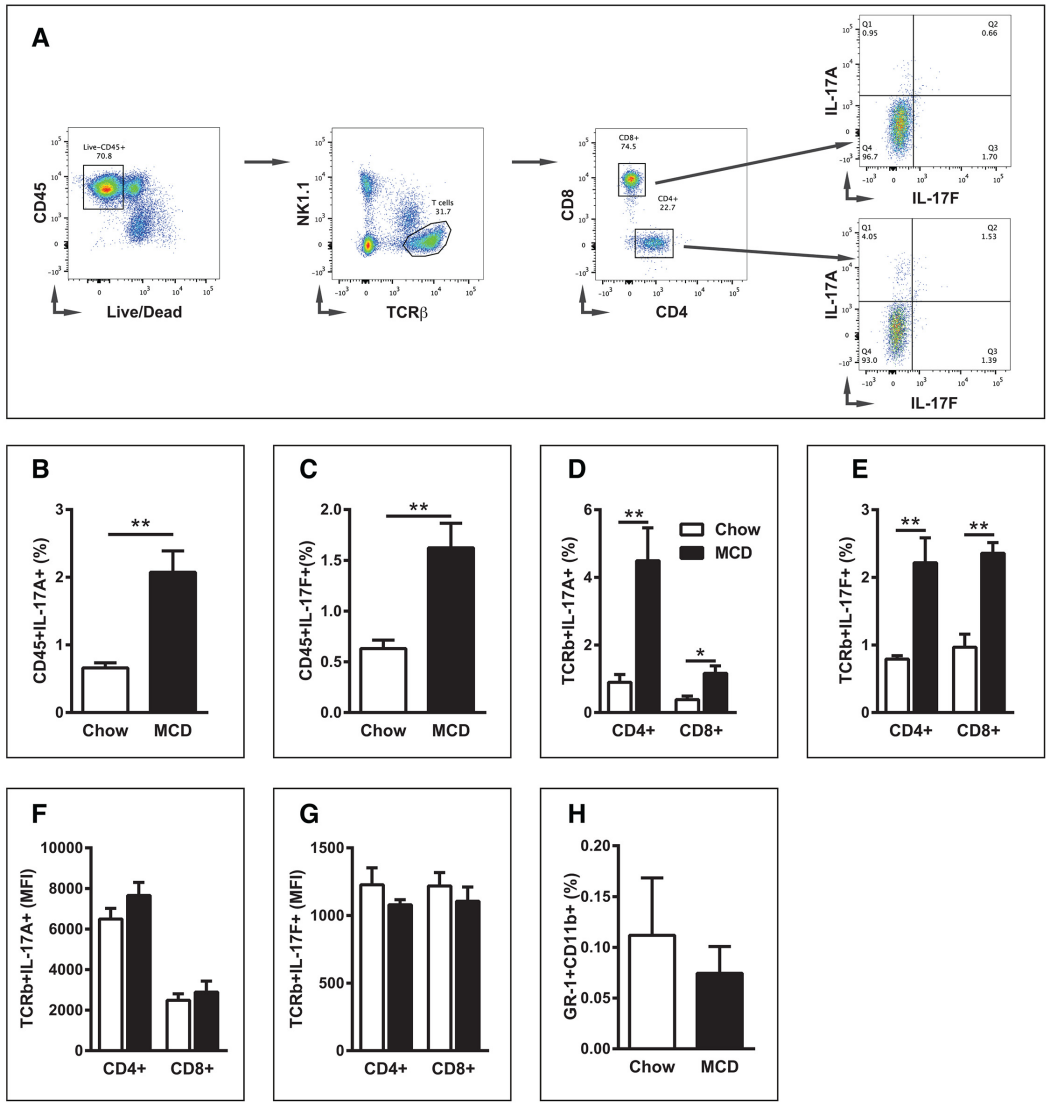
MCDD-driven NAFLD alters hepatic levels of IL-17A and IL-17F production. 8 week old male C57BL/6 mice (*n* = 4/condition) were placed on MCDD or chow diet for 4 weeks. (**A**) IL-17A and IL-17F production was determined in cells following the gating on: CD45^+^, TCRβ^+^, NK1.1^-^, CD4^+^ or CD8^+^. (**B**) Percent of hepatic immune cells (CD45^+^) producing IL-17A. (**C**) Percent of hepatic immune cells (CD45^+^) producing IL-17F. (**D**) Percent of hepatic TCRβ^+^ cells (CD4^+^ or CD8^+^ T cells) producing IL-17A. (**E**) Percent of hepatic TCRβ^+^ cells (CD4^+^ or CD8^+^ T cells) producing IL-17F. (**F**) Mean fluorescent intensity of IL-17A in TCRβ^+^ cells (CD4^+^ or CD8^+^ T cells). (**G**) Mean fluorescent intensity of IL-17F in TCRβ^+^ cells (CD4^+^ or CD8^+^ T cells). (**H**) Percent of hepatic immune cells (CD45^+^) expressing GR-1 and CD11b. Data represent means + SE; a representative of two separate experiments. Student *t* test **P < 0.01.

In light of increased IL-17RA expression, we next determined whether MCDD feeding altered IL-17A and IL-17F production in the liver. As shown in Fig 2, MCDD feeding led to an increase in hepatic infiltration of hematopoietic cells capable of producing both IL-17A and IL-17F (Fig 2A–2C). Specifically, numbers of both CD4^+^ and CD8^+^ T cells, cells known to robustly produce IL-17A and IL-17F, were found to be significantly upregulated. (Fig 2D and 2E) [15, 25]. However, the level of IL-17A and IL-17F production per cell, as measured by mean fluorescent intensity (MFI), was found to be unaltered. These data suggest that MCDD feeding drives an increased influx of IL-17RA ligand producing cells without augmenting IL-17A or IL-17F production on a per cell basis (Fig 2F and 2G). Of note, it has been shown that MCDD is associated with increased hepatic CD4^+^ T cell recruitment [26]. Further, both CD4^+^ and CD8^+^ T cells are thought to play substantial roles in NAFLD progression, and human NASH is characterized by infiltration of such cells to the liver [2]. Further, as IL-17 production is known to regulate tissue neutrophil infiltration [27], we next examined the impact of short term MCDD feeding on hepatic neutrophil influx. Notably, no significant difference in neutrophil infiltration was observed during MCDD feeding (Fig 2H). Thus, increased hepatic expression of IL-17A, IL-17F and IL-17RA suggested that IL-17 axis might play a role in regulation of MCDD-driven NAFLD pathogenesis.

### IL-17 axis deficiency increases steatosis, but protects from hepatocellular damage

**Fig 3:**
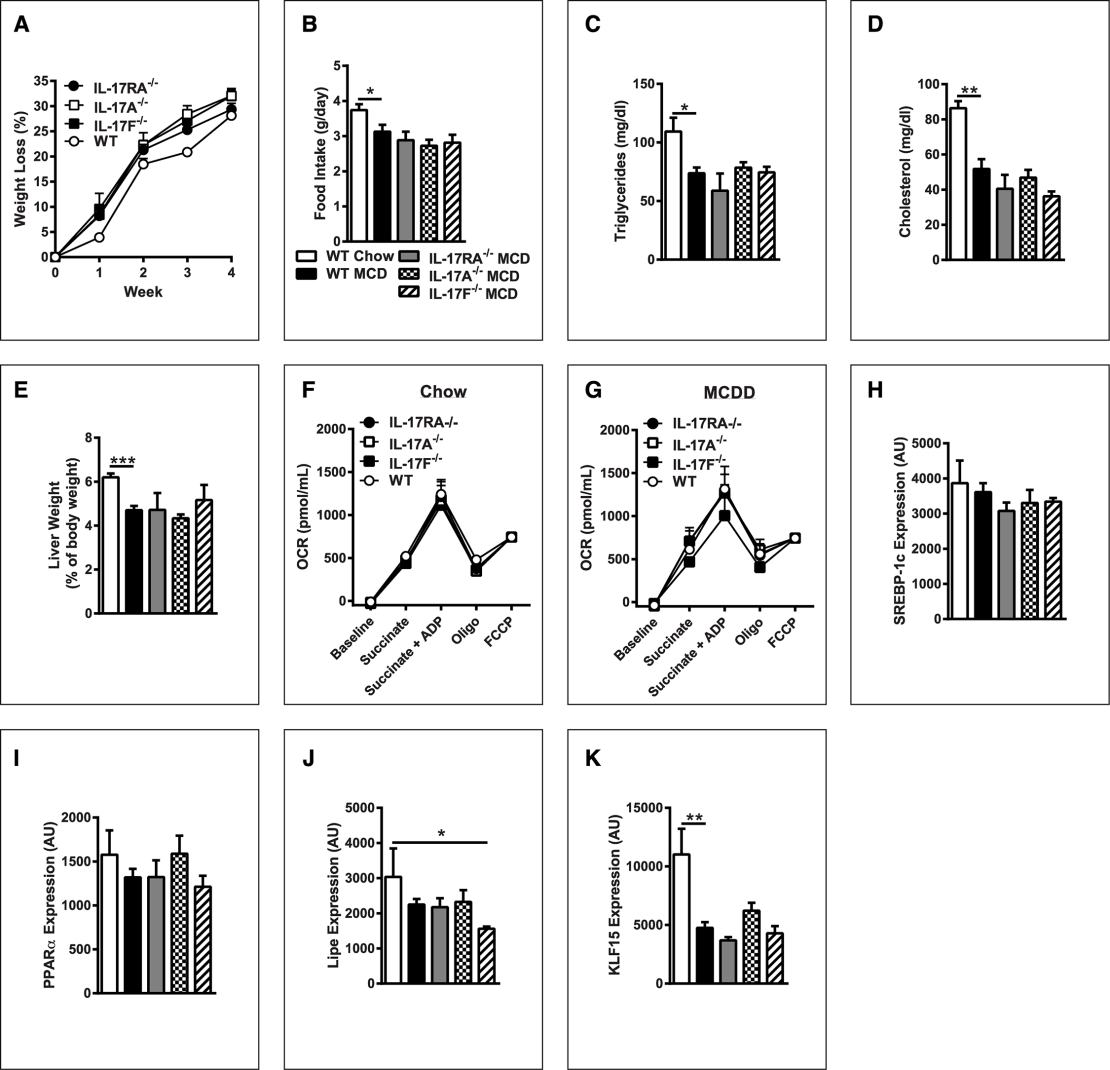
IL-17 axis deficiency does not modulate systemic effects of MCDD feeding. 8 week old male IL-17RA^-/-^, IL-17A^-/-^, and IL-17F^-/-^ mice on a C57BL/6 background and WT controls (*n* = 4-8/condition) were placed on MCDD or chow diet for 4 weeks. (**A**) Weight loss as percent of starting body weight. (**B**) Total daily food intake. (**C**) Serum triglyceride levels. (**D**) Serum cholesterol levels. (**E**) Liver weight as a percentage of total body weight. (**F**) Hepatic mitochondrial oxygen consumption rate in mice fed chow. (**G**) Hepatic mitochondrial oxygen consumption rate in mice fed MCDD. (**H**) Hepatic SREBP-1c mRNA expression. (**I**) Hepatic PPARα mRNA expression. (**J**) Hepatic Lipe mRNA expression. (**K**) Hepatic KLF15 mRNA expression. AU = Arbitrary Units, as compared to beta actin. Data represents means + SE; a representative of two separate experiments. Student *t* test *P < 0.05, ** *P* < 0.01, *** *P* < 0.001.

To define whether the increased hepatic IL-17RA expression and IL-17A and IL-17F production contributes to MCDD-driven NAFLD pathogenesis, WT, IL-17RA^-/-^, IL-17A^-/-^ and IL-17F^-/-^ mice were challenged with MCDD. No difference in body weight or food consumption was observed for mice fed a chow diet (data not shown). Although MCDD-feeding did induce weight loss, notably, IL-17RA^-/-^, IL-17A^-/-^ and IL-17F^-/-^ mice exhibited similar weight loss and food consumption as WT controls (Fig 3A and 3B). In addition, no differences were observed in other common hallmarks of MCDD stress, including serum triglyceride and cholesterol levels (Fig 3C and 3D) [24]. Moreover, the liver to body weight ratio among WT, IL-17RA^-/-^, IL-17A^-/-^ and IL-17F^-/-^ mice fed MCDD or fed chow diet was found to be similar (Fig 3E and data not shown). Further analysis also revealed no differences in mitochondrial respiration between chow and MCDD fed WT, IL-17RA^-/-^, IL-17A^-/-^ and IL-17F^-/-^ mice (Fig 3F and 3G).

In addition to hepatic mitochondrial function, NAFLD is associated with alterations of lipogenic pathways. Notably, MCDD feeding results in suppression of lipogenic pathways and blockade of lipoprotein export from the liver, and, as such does not constitute an ideal model to study lipogenic pathways in NAFLD. However, previous reports have demonstrated that inflammatory mediators associated with activation of IL-17 axis, IL-6 specifically, can regulate NAFLD pathogenesis in part via regulation of lipogenic machinery [28]. Thus, we evaluated whether lipogenic pathways were altered by IL-17 axis deficiency in mice fed MCDD. No differences were observed amongst MCDD-fed mice in hepatic mRNA expression of SREBP-1c, PPARα, or LIPE, potential markers of lipogenesis and lipid oxidation (Fig 3H–3J)[29]. In addition, hepatic levels of KLF15 were also unaltered in MCDD-fed mice (Fig 3K). Notably, IL-17A has been shown to regulate KLF15’s ability to augment triglyceride accumulation. Together, these data suggest that IL-17 axis does not alter lipogenic pathways in context of MCDD feeding [29, 30].

**Fig 4:**
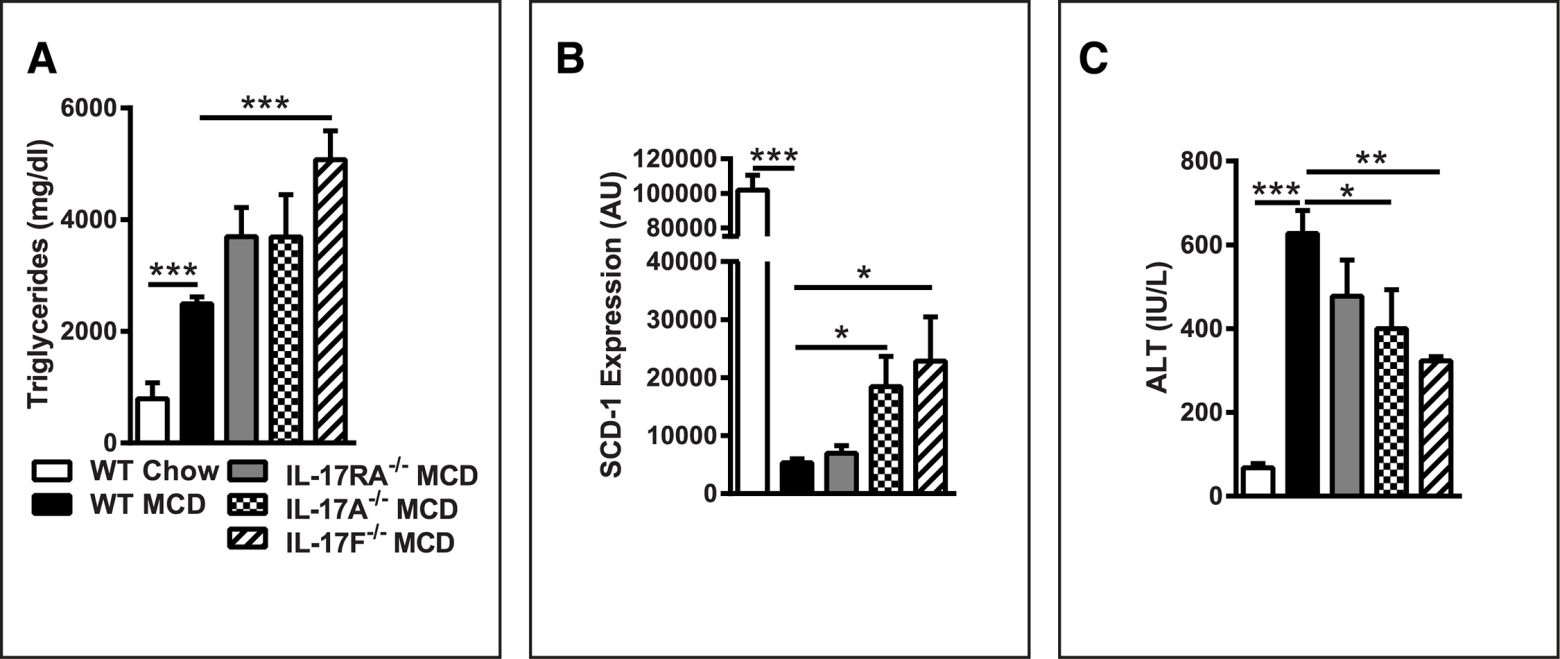
IL-17 axis deficiency augments hepatic steatosis, but protects from hepatocellular damage. 8 week old male IL-17RA^-/-^, IL-17A^-/-^, and IL-17F^-/-^ mice on a C57BL/6 background and WT controls (*n* = 4-8/condition) were placed on MCDD or chow diet for 4 weeks. (**A**) Hepatic triglyceride levels. (**B**) Hepatic SCD-1 mRNA expression. (**C**) Serum alanine transaminase (ALT) levels. AU = Arbitrary units, as compared to beta actin. Data represent means + SE; a representative of two separate experiments. Student *t* test *P < 0.05, ** *P* < 0.01, *** *P* < 0.001.

However, despite these similarities, MCDD-fed IL-17RA^-/-^, IL-17A^-/-^ and IL-17F^-/-^ mice exhibited increased levels of hepatic steatosis compared to WT mice, as determined by hepatic triglyceride deposition (Fig 4A). This increase in steatosis was found to correlate with hepatic stearoyl-CoA desaturase-1 (SCD-1) expression, an enzyme central to lipid metabolism (Fig 4B). Notably, SCD-1 deficient mice exhibit decreased steatosis, but increased hepatocellular damage during MCDD feeding [31]. Thus, these data suggest IL-17 axis plays a role in MCDD-driven NAFLD progression and hepatic steatosis.

To ascertain whether augmented hepatic steatosis and SCD-1 expression associated with IL-17 axis deficiency correlated with differential hepatocellular damage, serum ALT levels were quantified. Despite increased liver hepatic triglyceride accumulation when fed MCDD, IL-17RA^-/-^, IL-17A^-/-^ and IL-17F^-/-^ mice were protected from hepatocellular damage as compared to WT mice (Fig 4C). Animals fed chow diet displayed similar ALT levels (data not shown). These data suggest that a lack of IL-17RA, IL-17A or IL-17F uncouples steatosis from hepatocellular damage during MCDD-driven, obesity-independent, NAFLD and propose a novel role for IL-17F during disease pathogenesis.[3].

### IL-17 axis signaling regulates T cell infiltration to the liver

**Fig 5:**
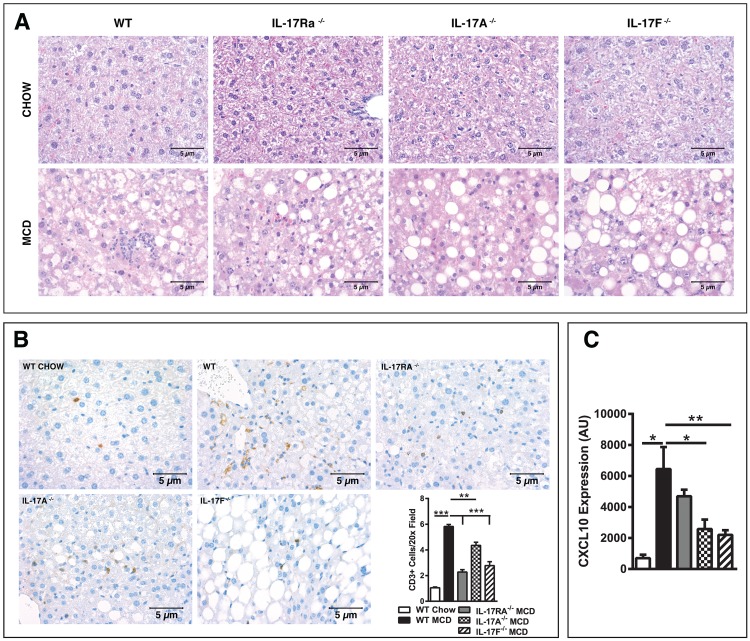
IL-17 Axis signaling regulates T cell infiltration to the liver. 8 week old male IL-17RA^-/-^, IL-17A^-/-^, and IL-17F^-/-^ mice on a C57BL/6 background and WT controls (*n* = 4-8/condition) were placed on MCDD or chow diet for 4 weeks. (**A**) Representative liver histology (H&E staining; 40x). Marked difference in the level and type of steatosis and inflammation between WT and IL-17RA^-/-^, IL-17A^-/-^ and IL-17F^-/-^ mice on MCDD. No differences were noted between genotypes on a chow diet. (**B**) Representative immunohistochemistry staining of CD3^+^ T cells in the livers of WT chow, along with WT, IL-17RA^-/-^, IL-17A^-/-^ and IL-17F^-/-^ mice on MCDD and average number of CD3^+^ cells per 20x field. (**C**) Hepatic CXCL10 mRNA expression. AU = Arbitrary units, as compared to beta actin. Data represents means + SE; a representative of two separate experiments. Student *t* test *P < 0.05, **P < 0.01, ***P < 0.001.

While the biological processes and mechanisms underlying the progression from steatosis to NASH are not fully understood, it is well accepted that augmented hepatic immune cell infiltration and production of immune mediators (proinflammatory cytokines and chemokines) play a central role in NAFLD pathogenesis. Of note, the MCDD is known to robustly upregulate hepatic inflammation [32]. To determine potential inflammatory pathways through which the IL-17 axis regulates progression from steatosis to steatohepatitis, we next examined severity of immune cell infiltration under MCDD stress. Histological analysis revealed that WT mice fed MCDD exhibited occasional hepatic lipidosis along with hepatic inflammation that ranged from medium to high levels throughout (Fig 5A). In contrast, IL-17RA^-/-^, IL-17A^-/-^ and IL-17F^-/-^ mice fed MCDD primarily displayed centrilobular, macrovesicular hepatic lipidosis with mild to moderate levels of hepatic inflammation based on H&E staining. These observations are in agreement with hepatic triglyceride levels. These data further support our observation that IL-17 axis uncouples steatosis and steatohepatitis in the context of MCDD stress (Fig 5A). Notably, no differences in steatosis, inflammation or immune cell infiltration were observed across genotypes in mice fed chow diet (Fig 5A).

NAFLD pathogenesis is associated with the infiltration of a variety of immune cells [2]. A more targeted analysis of hepatic infiltrating immune cells via immunohistochemistry revealed that IL-17RA^-/-^, IL-17A^-/-^ and IL-17F^-/-^ mice were specifically protected from the CD3^+^ T cell infiltration seen in WT mice on MCDD (Fig 5B). Notably, infiltration of T cells to the liver was demonstrated in MCDD-driven models of NAFLD and was shown to correlate with human NAFLD progression [2, 26]. In addition, recent evidence suggests that a T cell recruiting chemokine, CXCL10, plays an important role in MCDD-driven NAFLD progression [33]. While previous studies have demonstrated that IL-17A signaling directly induces CXCL10 production in esophageal cell lines [34], and the production of IL-17F is necessary for CXCL10 expression in hepatic viral infection models [35], whether the IL-17 axis induces CXCL10 during NAFLD remains undefined. Notably, IL-17RA^-/-^, IL-17A^-/-^ and IL-17F^-/-^ mice fed MCDD, but not chow diet, exhibited significantly reduced expression of CXCL10 compared to WT controls (Fig 5C and data not shown). Thus, IL-17 axis activation during MCDD-driven NAFLD pathogenesis may regulate both CXCL10 production and hepatic T cell infiltration; however, whether T cells or resident hepatic cells produce CXCL10 in this context remains undefined.

### The IL-17 axis regulates hepatic macrophage infiltration and downstream inflammation

**Fig 6:**
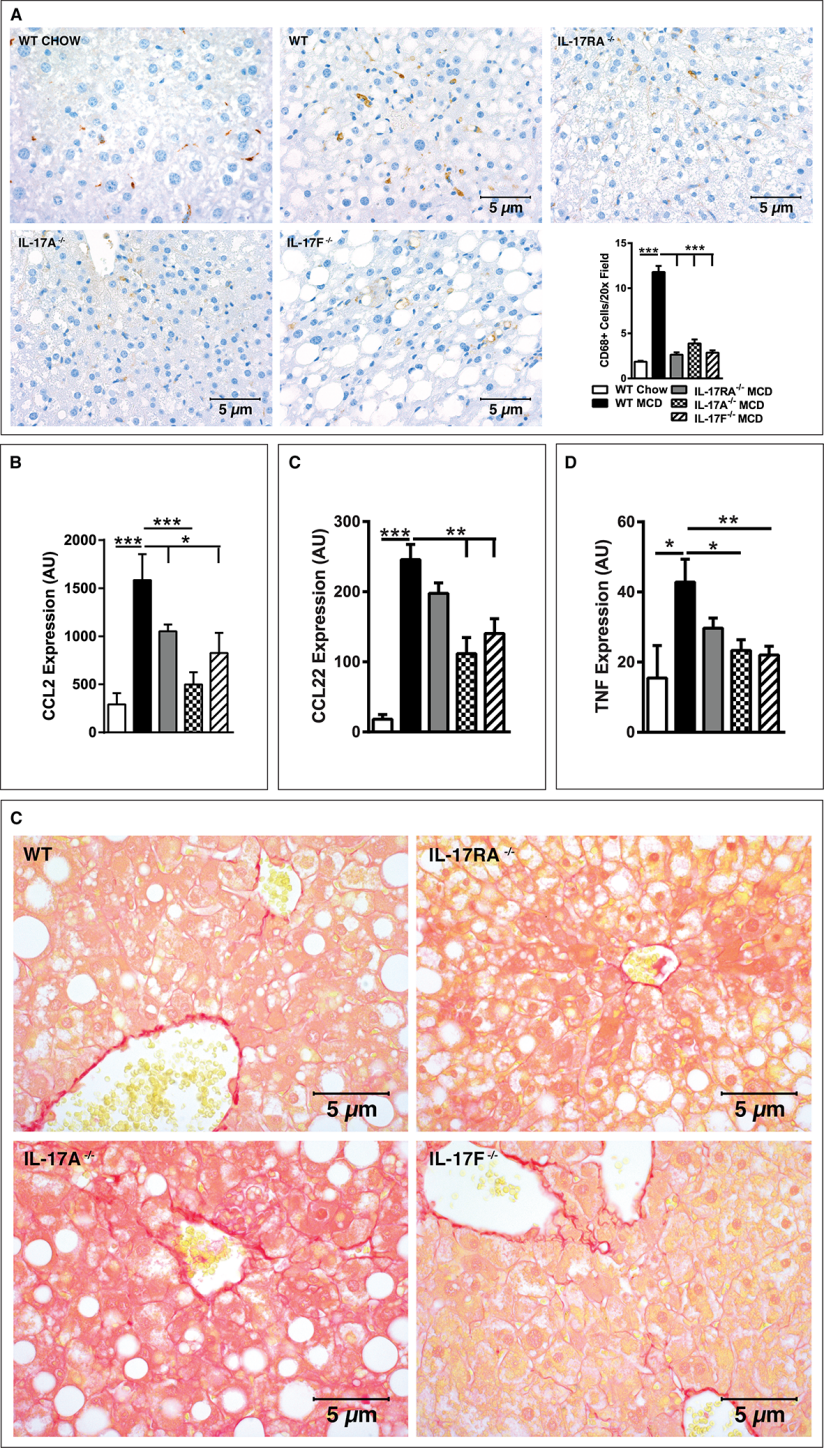
The IL-17 axis regulates macrophage infiltration to the liver. 8 week old male IL-17RA^-/-^, IL-17A^-/-^, and IL-17F^-/-^ mice on a C57BL/6 background and WT controls (*n* = 4-8/condition) were placed on MCDD or chow diet for 4 weeks. (**A**) Representative immunohistochemistry staining of CD68^+^ macrophages in the livers of WT mice fed chow and WT, IL-17RA^-/-^, IL-17A^-/-^ and IL-17F^-/-^ mice on MCDD and average number of CD68^+^ cells per 20x field. (**B-D**) Hepatic mRNA expression: (**B**) CCL2; (**C**) CCL22; (**D**) TNFα. (**E**) Representative Sirius Red staining of the livers from WT, IL-17RA^-/-^, IL-17A^-/-^, and IL-17F^-/-^ mice fed MCDD. AU = Arbitrary units, as compared to beta actin. Data represents means + SE; a representative of two separate experiments. Student *t* test *P < 0.05, **P < 0.01, ***P < 0.001.

In addition to hepatic T cell infiltration, infiltration of macrophages and their ability to produce proinflammatory cytokines is a hallmark of NAFLD-associated inflammation[2]. Further, increased hepatic macrophage numbers have been shown to correlate with NASH progression [2]. Therefore, we next quantified the number of hepatic CD68^+^ macrophages in context of MCDD feeding. As with CD3^+^ T cells, IL-17RA^-/-^, IL-17A^-/-^ and IL-17F^-/-^ mice exhibited a significant reduction in hepatic macrophage recruitment following MCDD feeding as compared to WT mice (Fig 6A).

Hepatic macrophage recruitment during NAFLD has been shown to be mediated by CCL2 production, a potent macrophage chemokine shown to be produced in response to CXCL10 expression [2, 33, 36]. As demonstrated above (Fig 1C and 1D), MCDD feeding robustly upregulates CD11b+F4/80+ macrophage infiltration and hepatic TNF expression in WT mice. However, whether the IL-17 axis impacts CCL2 expression in obesity-independent NAFLD remains unexamined. As shown in Fig 6B, MCDD-fed IL-17RA^-/-^, IL-17A^-/-^ and IL-17F^-/-^ mice had significantly reduced levels of hepatic CCL2 as compared to WT controls. No differences in hepatic CCL2 expression were observed in chow fed animals (data not shown). To expand on potential pathways through which the IL-17 axis regulates macrophage recruitment, we next examined hepatic CCL22 levels, a macrophage activating chemokine which also serves as a potential marker for human NASH [33, 37]. Of note, CXCL10 has also been implicated in regulation of CCL22 levels during MCDD-driven NAFLD progression [33]. As shown in Fig 6C, hepatic CCL22 expression was significantly reduced in IL-17RA^-/-^, IL-17A^-/-^ and IL-17F^-/-^ mice as compared to WT mice when fed MCDD, but not chow diet (data not shown). We next examined whether the IL-17 axis’s regulation of macrophages altered hepatic cytokine production. Specifically, TNF production, at least in part, is regulated through CXCL10, and has been shown to augment MCDD-driven hepatic inflammation [2, 33, 38]. As shown in Fig 6D, IL-17RA^-/-^, IL-17A^-/-^ and IL-17F^-/-^ mice, compared to WT controls, were protected from MCDD-driven increase in hepatic TNF expression. Moreover, no difference in hepatic expression of TNF was found in chow fed animals (data not shown). These data suggest that the IL-17 axis regulates recruitment of macrophages to the liver during MCDD-driven NAFLD. Further, our findings suggest that IL-17 axis-driven modulation of CXCL10 and its downstream pathways may play a significant role in regulation of NAFLD progression.

Further, increased hepatic immune cell infiltration and proinflammatory marker expression is associated with the development of fibrosis. Similarly, IL-17 axis has been shown to play a role in induction of fibrosis [39]. Thus, we next examined whether the IL-17 axis affects fibrosis in in the context of short-term MCDD feeding. As shown in Fig 6E, short term MCDD feeding did not induce fibrosis in any genotype. These findings are not surprising as previous reports have demonstrated that long-term MCDD feeding is required to induce hepatic fibrosis [40–42]. Thus, our data suggest that the early effect of IL-17 axis-driven modulation of hepatocellular damage occur independently of the modulation of fibrosis and that further studies involving long term MCDD feeding are required to formally define the role of IL-17 axis in development of fibrosis and its impact on hepatocellular damage.

## Discussion

NAFLD is the most common liver disorder in the world [1] and is expected to surpass Hepatitis C infection as the number one cause for requiring liver transplant [43]. Despite the clinical significance, specific therapies for NAFLD are lacking. Although the etiology of NAFLD is under-defined and certainly multifactorial, it is well accepted that inflammation is a central component to NAFLD development and progression [2]. Thus, a better understanding of the immune pathways underlying NAFLD pathogenesis may be critical to developing novel strategies for therapeutic intervention. Taken together, our data provide a novel insight into the role of IL-17 axis-mediated regulation of inflammation during NAFLD progression. Specifically, our data provide initial evidence that (**a**) IL-17F signaling also plays a critical role in NAFLD pathogenesis and (**b**) the IL-17 axis plays a broad role in multiple models of NAFLD via modulation of hepatic inflammation.

While IL-17RA, in part via IL-17A, was previously determined to regulate obesity-dependent NAFLD, our novel data show that hepatic IL-17RA expression is actually increased during MCDD-induced NAFLD [3]. Further, while immune cells ubiquitously express IL-17RA [15], expression of IL-17RA was not found to be upregulated in hepatic immune cells. However, whether or not immune cells in the NAFLD setting are more responsive to IL-17 axis signaling remains to be determined. Further, it is possible that resident liver IL-17RA expressing cells play a critical role in disease progression during MCDD-driven NAFLD progression, given that their expression of IL-17RA is increased. Notably, among resident hepatic cells, both hepatic stellate cells and hepatocytes have been shown to express IL-17RA and are known to activate inflammatory pathways which exacerbate disease [2]. While uncovering the critical IL-17RA expressing cell type(s) in NAFLD would greatly aid in understanding the IL-17 axis’s role in disease, such findings would likely require the development and utilization of novel experimental tools, including cell type specific knockout mice. Additionally, although MCDD-driven NAFLD occurs through the inhibition of beta-oxidation and even drives weight loss, investigation of the mechanisms driving increased IL-17RA expression during MCDD stress may allow for a better molecular understanding of the activation of inflammation in NAFLD pathogenesis.

The IL-17A and IL-17F producing cell type(s) during NAFLD have not been previously evaluated. Our novel data show that hepatic CD4^+^ and CD8^+^ T cells are major producers of IL-17A and IL-17F in the context of MCDD-driven NAFLD. While a role for CD8^+^ T cell production of IL-17A or IL-17F has not been previously described in hepatic diseases, CD4^+^ T cells (Th17 cells) are known to be substantial producers of hepatic IL-17 levels [10, 26]. Recent evidence suggests that the Th17 cell population is heterogeneous, consisting of both pro- and anti-inflammatory subsets, which can play opposing roles through differential cytokine production [44]. In a variety of diseases, including Crohn’s disease, multiple sclerosis, and rheumatoid arthritis, levels of pro-inflammatory Th17 cells are known to be highly upregulated in the inflamed tissues compared to peripheral blood [44]. However, whether a specific Th17 subset has a dominant role in the liver during NAFLD progression remains undefined. Additionally, IL-17A and IL-17F production is not limited to CD4^+^ and CD8^+^ T cells. Other cells capable of producing IL-17A and IL-17F include NKT cells, γδ T cells and ILC3s [35, 45]. Thus, determining the critical cytokine producing cell(s) will require further investigation, likely involving specific knockouts or cell-specific transfer studies into IL-17A- and IL-17F-deficient mice. Although it is likely that multiple cells contribute to the increased IL-17A and IL-17F production observed during NAFLD pathogenesis, the identification of the major hepatic IL-17RA ligand producers may result in specific treatment options that do not compromise the overall systemic IL-17 response.

While a role for IL-17A in obesity-related diseases has been previously described [3, 46], the role demonstrated herein for IL-17F is completely novel. The requirement for both IL-17A and IL-17F in NAFLD progression described above provides novel insights into the mechanisms through which they mediate disease progression. The fact that deficiency of either IL-17A or IL-17F protects from disease progression suggests that IL-17RA activation by both cytokines is required for disease pathogenesis. In fact, such findings may indicate that the heterodimer IL-17A/F has a unique and critical role in NAFLD progression. In fact, the heterodimeric complex of IL-17A/F has been shown to regulate inflammatory responses [47]. However, our results may also demonstrate that IL-17A and IL-17F act in epistasis, and possibly argue that both pathways must be engaged for NAFLD pathogenesis–something that may occur in parallel and/or in multiple cell types. Moreover, IL-17A^-/-^ and IL-17F^-/-^ mice seem to exhibit a more robust protection from hepatic inflammation and hepatocellular damage than IL-17RA^-/-^ mice (Fig 4C). These data further suggest that IL-17RA signaling may also play a protective role in NAFLD via indirect pathways. In fact, a protective role for IL-17RA in NAFLD progression was recently shown to be dependent on IL-17E signaling [48]. Additionally, other IL-17 family receptors, and specifically IL-17RC, may play a role in NAFLD pathogenesis. Lastly, a lack of either IL-17A or IL-17F may be sufficient for an increase in anti-inflammatory and/or immune-regulatory mediators normally inhibited by the IL-17 axis cytokines, such as T regulatory cell expansion and activation, and this accrual could be responsible for protection from NAFLD progression [26].

Further, our data also imply that IL-17 axis activation may be an initial, or early, hit in NAFLD progression. Specifically, our data argue that a lack of IL-17 axis activation leads to decreased downstream production of inflammatory mediators known to drive NAFLD; including, CXCL10, CCL2, CCL22, and TNF. While esophageal cells have been shown to upregulate CXCL10 after IL-17 stimulation [34], whether IL-17A and IL-17F directly upregulate CXCL10 during NAFLD progression remains unexamined. However, reliably demonstrating the presence and necessity of this mechanism will likely depend on first determining the critical IL-17RA expressing cell type(s).

In sum, this report further implicates IL-17RA in NAFLD pathogenesis and provides initial evidence for IL-17A and IL-17F as significant contributors to MCDD-driven disease progression. Specifically, MCDD-driven NAFLD is associated with increased hepatic IL-17RA expression and IL-17A/IL-17F production. A deficiency of IL-17RA, IL-17A or IL-17F protects from increased proinflammatory cytokine and chemokine production, immune cell infiltration and hepatocellular damage. Further, the role for IL-17 axis in multiple experimental models of NAFLD, including high fat diet, high fat high carbohydrate diet and MCDD, strongly suggests that the IL-17 axis may represent an amicable therapeutic target for the rising numbers of NAFLD cases [1, 49]. Notably, multiple clinical trials are currently employing the use of IL-17A and IL-17RA antibodies for a variety of diseases [27]. Targeting either of these, or even IL-17F or IL-17A/F heterodimeric complex, may prove a useful method to treating the growing NAFLD incidence.
